# Perspectives of Trial Staff on the Barriers to Recruitment in a Digital Intervention for Psychosis and How to Work Around Them: Qualitative Study Within a Trial

**DOI:** 10.2196/24055

**Published:** 2021-03-05

**Authors:** Stephanie Allan, Hamish Mcleod, Simon Bradstreet, Imogen Bell, Helen Whitehill, Alison Wilson-Kay, Andrea Clark, Claire Matrunola, Emma Morton, John Farhall, John Gleeson, Andrew Gumley

**Affiliations:** 1 University of Glasgow Glasgow United Kingdom; 2 Orygen Melbourne Australia; 3 University of British Columbia Vancouver, BC Canada; 4 La Trobe Melbourne Australia; 5 Australian Catholic University Melbourne Australia

**Keywords:** recruitment, schizophrenia, mHealth, psychosis, mental health

## Abstract

**Background:**

Recruitment processes for clinical trials of digital interventions for psychosis are seldom described in detail in the literature. Although trial staff have expertise in describing barriers to and facilitators of recruitment, a specific focus on understanding recruitment from the point of view of trial staff is rare, and because trial staff are responsible for meeting recruitment targets, a lack of research on their point of view is a key limitation.

**Objective:**

The primary aim of this study was to understand recruitment from the point of view of trial staff and discover what they consider important.

**Methods:**

We applied pluralistic ethnographic methods, including analysis of trial documents, observation, and focus groups, and explored the recruitment processes of the EMPOWER (Early Signs Monitoring to Prevent Relapse in Psychosis and Promote Well-being, Engagement, and Recovery) feasibility trial, which is a digital app–based intervention for people diagnosed with schizophrenia.

**Results:**

Recruitment barriers were categorized into 2 main themes: service characteristics (lack of time available for mental health staff to support recruitment, staff turnover, patient turnover [within Australia only], management styles of community mental health teams, and physical environment) and clinician expectations (filtering effects and resistance to research participation). Trial staff negotiated these barriers through strategies such as emotional labor (trial staff managing feelings and expressions to successfully recruit participants) and trying to build relationships with clinical staff working within community mental health teams.

**Conclusions:**

Researchers in clinical trials for digital psychosis interventions face numerous recruitment barriers and do their best to work flexibly and to negotiate these barriers and meet recruitment targets. The recruitment process appeared to be enhanced by trial staff supporting each other throughout the recruitment stage of the trial.

## Introduction

### Background

To better understand how interventions could be developed, evaluated, and implemented in routine care, it is important to fully understand which aspects of the implementation of randomized control trials (RCTs) are most challenging [1]. All RCTs must recruit participants for interventions to be tested [2]. However, recruitment into RCTs can be very difficult and is possibly the biggest challenge within clinical research [3], with many RCTs failing to reach their recruitment targets [4]. Delayed recruitment can lead to additional costs [5], and underpowered clinical trials can threaten the empirical value of intervention research [6]. Systematic reviews of recruitment barriers have helped uncover specific barriers for recruiting ethnic minority populations [7], within HIV trials [8] and cancer trials [9]. However, reviews are only possible if primary data are collected and shared. Digital interventions are becoming popular for increasing access to treatments; however, little is known about the nature of specific recruitment barriers in these trials [10]. Beyond widespread societal concern about the negative impacts of digital technology within daily life [11], there may be recruitment challenges in mental health care research, such as concerns that patients may struggle to use a digital device [12]. However, systematic review evidence suggests that these effects are not yet understood because trial recruitment is not covered in depth in studies of implementation barriers for digital interventions for psychosis [13].

Trial staff responsible for recruiting participants must implement something novel (in this case, the recruitment process for a new intervention) within a health care system that comes with existing norms, knowledge, and social practices. Trial recruitment involves interacting with diverse groups [14] including patients, clinical staff, clinical leaders, and other members of the trial team. The health care system can be described as a context in which the recruitment process must fit. Process evaluations use qualitative research to develop an understanding of how trial processes such as recruitment were delivered and received by participants and trial staff [15,16]. Context in process evaluation terms is defined as factors external to an intervention that influence clinical trial processes’ delivery [17], such as recruitment. Therefore, understanding the context of recruitment is important for understanding what factors may act as barriers and facilitators in enrolling participants within a clinical trial.

Use of and interest in digital interventions is high in people diagnosed with schizophrenia [18], and digital interventions for psychosis are growing in popularity [19,20]. Currently, the ongoing COVID-19 pandemic has seen a surge in interest in using digital technologies to support people with mental health problems [21]. However, the willingness of patients to be recruited into digital intervention clinical trials is poorly understood [22,23]. People diagnosed with schizophrenia are described as a difficult-to-recruit population, more generally within clinical trials [24]. Recruitment for service users diagnosed with schizophrenia often involves approaching patients via staff; therefore, it seems particularly important to consider the role of staff within study recruitment. For example, a recent study reported that 1 in 5 mental health staff report having never recruited a service user into a research study [25].

Within trials of digital interventions, it is recommended that the recruitment of end users should be described in sufficient detail to enable readers who wish to contextualize or replicate the work [26]. Feasibility studies help establish important parameters such as the willingness of clinicians to recruit patients and the willingness of participants to be randomized [27]. Despite the importance of recruitment, CONSORT (Consolidated Standards of Reporting Trials) statements [28] do not require RCT reporting to describe recruitment in detail beyond documentation of participant flow [29,30]. The proposed CONSORT extensions [31] recommended that qualitative data be collected so that context can be more fully understood and so that future researchers may recognize relevant contextual elements (such as settings and stakeholder participation) that are necessary for the replication of findings observed within a particular trial. Reporting a more detailed examination of recruitment processes, particularly recruitment barriers [32], is suggested to be useful in interpreting trial results and developing strategies for improvement [33]. Moreover, failure to report recruitment experiences risks significant loss of a key source of knowledge. In addition, it is important to note that detailed reporting of recruitment into digital intervention studies using mobile apps is scarce [34].

Trial staff are responsible for meeting recruitment targets, which requires interacting with potential participants. This places them in a unique position to comment on the overall recruitment process and provides a narrative on (1) what happened during trial recruitment and (2) to enable researchers to make informed comment on why. Identifying barriers to recruitment has been identified as a strength of qualitative research within clinical trials [35,36]. Furthermore, qualitative research could also describe what strategies trial staff use to negotiate around recruitment barriers. However, to the best of our knowledge, there is little empirical exploration of the trial recruitment process directly from the point of view of trial staff.

### Study Aims

This qualitative study within a trial (SWAT) [37] aimed to gather and analyze data to more fully understand barriers and facilitators encountered by trial staff during the recruitment process for the EMPOWER (Early Signs Monitoring to Prevent Relapse in Psychosis and Promote Well-being, Engagement, and Recovery) study (described in more detail later) and to facilitate learning ahead of a full trial. Previous qualitative work conducted with carers, mental health staff, and service users suggested that recruitment barriers were hypothesized within the EMPOWER trial [12], such as service users feeling paranoid in response to digital technology and a lack of staff time to support the recruitment process. Therefore, this study aims to explore recruitment issues in some depth but was not limited to the a priori issues identified in our previous research.

EMPOWER [38] (ISRCTN: 99559262) aimed to develop and evaluate a mobile app for use with adults who experience psychosis. The EMPOWER app is a digital self-management tool (augmented with peer support) to enhance the identification of and communication about early warning signs of relapse in people diagnosed with schizophrenia. The app enables routine self-monitoring for a variety of different experiences, including psychosis (eg, hearing voices and suspicious thoughts), anxiety, mood, self-esteem, and interpersonal support. EMPOWER participants used the app for an initial 28-day baseline period to identify their typical variation in personal well-being. Significant changes from baseline are then triaged by a clinician, and, if necessary, mental health staff are notified. EMPOWER was tested in a cluster randomized control trial (cRCT). As EMPOWER was trying to enhance communication and shared decision making between multiple stakeholders, mental health staff, service users, and carers (if relevant) were all potential participants. The feasibility of the EMPOWER intervention and study procedures was tested in a multisite trial in both Australia and the United Kingdom. The initial recruitment target was 120 service user participants (and any linked carers) and 40 mental health staff from 8 community mental health services (CMHS) before randomization of the clusters (services). During the course of the study, 8 CMHS were recruited and randomized; however, a revised recruitment target of n=86 was agreed upon and met.

In cluster trials, outcomes are usually measured at the level of the individual; however, trial procedures (such as recruitment) are applied by the research team at the level of the cluster (in this case, adult community mental health teams) [39]. When recruitment for EMPOWER began, research assistants within EMPOWER electronically screened medical records of local CMHS for potentially eligible participants and then approached key workers employed within adult community mental health teams (the cluster) who had potentially eligible participants on their case load. Therefore, developing an understanding of recruitment both within and across sites appears important in contextualizing the recruitment process in a cRCT such as EMPOWER. Full details of the intervention are reported in the protocol [38]. In a feasibility study such as EMPOWER, process evaluators are usually interested in facilitators and barriers to implementation so that strategies to enhance implementation of key processes such as recruitment can be put in place for a definitive trial [17].

## Methods

### Theoretical Framework

In line with the EMPOWER process evaluation protocol [40], the theoretical framework for this study was constructivism [15], which posits that knowledge is created through social interactions. The processes that occur during intervention implementation need to be understood in ways that are responsive to the complexities and intricacies of programs, people, and places [41]. Recruitment in clinical trials is a complex social action; therefore, there is unlikely to be one definitive methodology (qualitative or otherwise) that can allow us to theorize recruitment in sufficient depth [42].

The primary focus of the analysis was on achieving the a priori study aims (understanding the context of recruitment during the feasibility trial stage to refine recruitment in a full trial). Particular attention was paid to the reporting of barriers and facilitators to recruitment because this helps understand the context of recruitment. We now describe the 2 methods of the study in line with the key aim.

### Ethnography

Ethnography refers to both the process and outcome of research that produces rich descriptions and interpretations of a social system from the point of view of its key social actors, including their behaviors, roles, and methods of interaction [43]. Ethnography is useful for theorizing implementation processes such as recruitment because ethnographic narratives pay attention to interconnectedness while building a holistic understanding of how systems come together as a whole [44,45]. Furthermore, ethnography is useful for developing internally valid theory by focusing on describing how people behave in the real-world context of clinical trial recruitment. Taking an ethnographic stance is advantageous in process evaluation research because it can help develop the implementation theory of key trial processes with good internal validity [46].

SA was based within the main office of EMPOWER for the full duration of trial recruitment and was able to observe trial staff both within meetings and within their daily office-based tasks during the recruitment process. Although ethnography commonly involves a researcher directly observing social processes, the examination of administrative data and study documents is important within process evaluation research [47]. Therefore, the minutes of team meetings were seen as sites for ethnographic inquiry beyond what SA recorded from observation. This was considered to be particularly useful because SA could not directly observe recruitment processes that occurred outside of the office.

### Trial Staff Focus Groups

To triangulate findings from the observation-based ethnography, focus groups were held with members of trial staff who were involved in the recruitment process. The use of qualitative methods [48] and, in particular, focus groups within an RCT facilitates the understanding of the recruitment process [49]. Exploring recruitment from the point of view of the trial staff who worked on the trial and who experienced the recruitment process directly is noted to be useful because it provides insight into the reasons behind what can be observed [35]. Ethics approval for this study was received from the West of Scotland Research Ethics Service (GN16MH271 Ref: 16/WS/0225) and Melbourne Health (HREC/17/MH/97 Ref: 2017.010).

### Procedure

#### Ethnography

SA (who was based in the UK office for the EMPOWER study) was present at the majority of weekly team meetings in the United Kingdom that were held during the recruitment process and had access to the minutes of meetings from this time. All members of the EMPOWER team who were based in Glasgow attended these meetings, with the focus of discussion being on general trial business. Recruitment procedures for both the United Kingdom and Australia were discussed in these meetings. Beyond formal meetings, SA was able to observe the work of the trial staff within the office and was privy to their discussions and reflections on the matter for the duration of trial recruitment. SA recorded reflective notes during the recruitment process from ethnographic observations at both formal meetings and more informal *daily work* and then consolidated these into reflective memos once the recruitment period was over. SA revisited meeting minutes (n=50) for the period from August 03, 2017, when recruitment started, to July 05, 2018, when the recruitment target was achieved (n=86), to refresh their memory and wrote reflective ethnographic memos. Relevant ethnographic reflections are reported in addition to analyses from the focus groups. Observational data from meeting recordings and field notes were anonymized.

#### Trial Staff Focus Groups

Both focus groups were facilitated by SA (independent of the research team). One focus group was facilitated in person in Glasgow, United Kingdom, and another was facilitated remotely with the Australian team in Melbourne, who participated remotely via a secure telephone interface. Verbal informed consent was obtained before the start of each focus group. Each focus group followed a schedule of questions designed to explore barriers and facilitators to recruitment in some depth. A semistructured interview schedule was developed for broad exploration of the recruitment process from the perspective of trial staff (schedule available in prepublished protocol [40]). Both focus groups were audio recorded and then transcribed verbatim. Focus groups lasted for an hour. All focus groups were held during the typical working day for trial staff, and participation was voluntary. Data have been anonymized to protect confidentiality; all participants are simply referred to as *Participant*, with numbers being used for clarity when a textual extract has data from more than one participant.

All participants in this SWAT (through observation or focus group participation or both) were employed in the EMPOWER trial and were involved in trial recruitment (either directly or indirectly). EMPOWER was a feasibility study; therefore, the numbers reflect the relatively small pool of trial staff, which is highlighted in Table 1. NVivo [50] software was used for all analyses.

**Table 1:** Description of participants’ characteristics.

Location	Focus group attendees	Roles
United Kingdom	6 (out of a possible 7)	Researcher, Chief Investigator, and Trial Manager
Australia	3 (out of a possible 5)	Principal Investigator, Researchers, and Trial Manager

#### Reflexivity

SA is a PhD student working on a process evaluation for the EMPOWER cRCT [38]. The PhD funding SA receives is independent of any funding associated with the trial. Following observations of trial staff during the recruitment process, it seemed as though the recruitment process was a key site of inquiry to more fully understand full trial feasibility. Therefore, a decision was made to undertake a small qualitative SWAT. Supervision and finalization of the coding process was done in conjunction with HM and AG, who are academic clinical psychologists, academic supervisors to SA, and investigators on the EMPOWER trial.

### Analysis

All data, including ethnographic observations and focus group transcripts, were analyzed thematically by SA using thematic analysis, a qualitative method used to identify, analyze, and report patterns constructed within text data [51]. The first stage comprised line-by-line coding (descriptive) moving onto the second stage of coding, where descriptive codes were thematically linked together into a final set of themes. Constructivist qualitative research assumes that themes do not emerge from data but are constructed as part of a reflexive analytic process [52]. Therefore, themes will be reported as being constructed. Trial staff provided critical feedback on the rigor and validity of the thematic analysis, similar to member checking [53].

## Results

**Figure 1:**
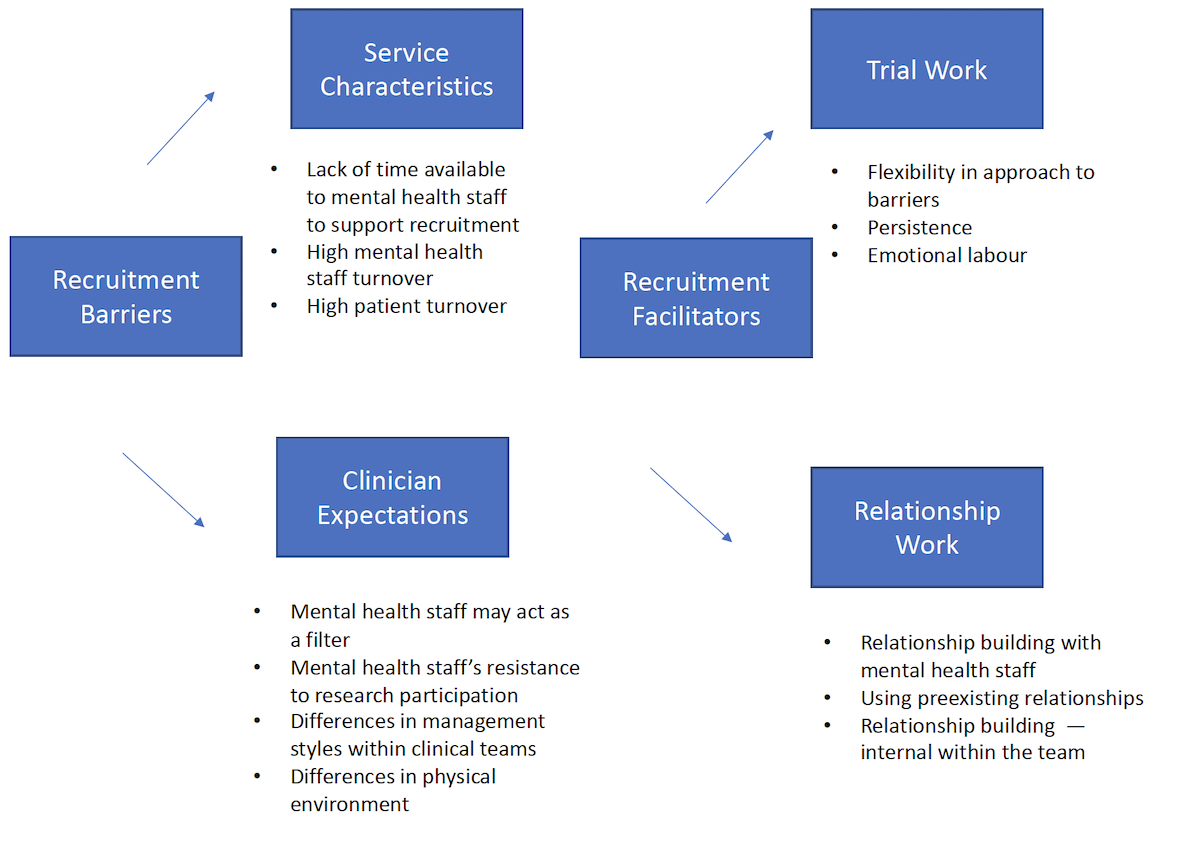
Thematic map of recruitment themes.

Following thematic analyses of ethnographic observations and focus groups, it seemed that there were several key recruitment barriers encountered by the research team during recruitment to the trial. Beyond simply listing recruitment issues, trial staff discussed how these issues were addressed and what work was done to best negotiate these issues. To frame these discussions as distinct from merely reporting key issues, the concept of *trial work* [54] was used within a qualitative framework analysis [55]. Trial work is a broad concept related to the work done to overcome barriers during the recruitment process engagement, *buy in* to the trial across a range of stakeholders, and work involved in managing the organizational complexity necessary to reach recruitment targets [54]. Trial work appeared to be highly relevant to the aims of this study in terms of maximizing learning and understanding from the EMPOWER recruitment process. The reporting will highlight the key *recruitment barriers* and then the *trial work* used to facilitate recruitment. We summarize the themes in Figure 1 and then describe the themes and provide portions of raw data to make the analysis more transparent.

### Key Recruitment Barriers

The key barriers described by trial staff into trial recruitment broadly fell into 2 main themes: service characteristics (lack of time available to mental health staff to support recruitment, staff turnover, patient turnover [within Australia only], management styles of community mental health teams, and physical environment) and clinician expectations (filtering effect and resistance to research participation).

#### Service Characteristics

##### Lack of Time Available to Mental Health Staff to Support Recruitment

Research trial staff frequently spoke about mental health staff not having much time to engage in the recruitment process. The research team was highly aware of the broader social context of low staff capacity in the face of high numbers of patient referrals in routine care with limited staff to meet demand. Trial staff at both sites made empathetic references to being aware of mental health staff working within a context of immense pressure with a lack of resources and support. During the analysis by SA, it was constructed that the trial staff in EMPOWER felt it was inevitable that structural barriers that lead to mental health staff not having much spare time would inevitably be a barrier to trial recruitment:

I don’t think you can relate how busy they are. And much pressure they’re under. Some of the numbers we heard about in terms of new referrals into teams were quite staggering.

Participant 1

Forty. Forty referrals a week, yeah. And there doesn’t seem to be any sort of throughput to accommodate that additional pressure being moved around.

Participant 2, United Kingdom

##### High Mental Health Staff Turnover

Closely linked to a lack of staff time was high staff turnover, which appeared to be systemic across both trial sites. Meeting notes and focus group data from both the United Kingdom and Australia indicated that high clinical staff turnover was a challenge to recruitment. Practically, this led to issues such as new clinical staff not being aware of the study because they were not employed when staff teams were initially told about it. Clinical staff changing jobs or taking leaves as they were unwell also appeared to be systemic issues within mental health services and was a macrolevel recruitment challenge. In the following example, a member of the EMPOWER team reflects on the impact of high staff turnover:

What we’re seeing is the key workers [mental health staff] are very fluid, there’s loads of movement, there’s massive changes as to who your key worker is, there’s lots of staff turnover.

Participant, United Kingdom

##### High Patient Turnover

A related subtheme (which was exclusive to Australia) was patient turnover because patients are discharged back to general practice (as evidenced in the quote below where participant alludes to “it’s not only a high turnover of consumers [patients]”) following the end of an acute episode of psychosis, unlike in the United Kingdom where clinical support is generally more long term for people diagnosed with schizophrenia. This was a particular barrier to recruitment because if patients were no longer in the service, they simply could not be recruited. However, this issue intersected with high clinical staff turnover, resulting in a complex barrier to recruitment into the study because the high clinical staff turnover within mental health services blocked the ability of trial staff to build relationships with clinical staff to build trust in the team and the project:

I think it's also worth noting that in public mental health services it's not only a high turnover of consumers [patients] but there's also a pretty high turnover of staff in some places, so you would have some clinicians that hadn’t heard of it or you know were quite new around that time and that kind of translates to recruiting consumers as well in terms of the discharges and the change in people being part of the service.

Participant, Australia

##### Differences in Management Styles Within Clinical Teams

In both the United Kingdom and Australia, there were discussions about differences in management styles between the different mental health teams. In the first example, a trial team member explicitly stated that although participant numbers between sites may not have appeared too different, this obscured the challenges of having to adapt to different leadership styles across mental health teams. This was viewed as a key determinant of recruitment success:

I think at the big picture level the rate of recruitment wasn't particularly different and you know, [other named research assistants] might be able to say a bit more about the style of how it happens etc., there are certainly very different personality styles of managers so in terms of us managing the managers, we had to take into account that there are very different people who had a very different styles.

Participant, Australia

However, as pointed out in the UK site, it was not always the case that managers were those who were *pulling the strings* in terms of creating barriers to recruitment:

Leadership’s hugely important in this. And always underestimated how much influence it has in any field, but this one no less. That the messages and the values and the attitudes that are being shared by the person who’s pulling the strings is really, really important. And that person who’s pulling the strings isn’t necessarily always the person who is supposed to be pulling the strings.

Participant, United Kingdom

##### Differences in Physical Environment

A further important recruitment challenge stemmed from the layout of the physical premises of mental health services themselves. Although this may be unique to a particular center, the impact upon recruitment was considered by trial staff to be large. For example, 2 researchers recalled the impact of the physical layout of premises, which hindered their ability to develop relationships with staff and acted as a significant block to successful social interactions:

The physical environment’s really problematic there [named recruitment site] as well, because they’re all in small, separate offices, so it doesn’t really feel like a team. So individual and...

Participant 1

There’s nowhere to circulate and to talk to the nurses.

Participant 2

There’s nowhere to chat amongst yourself, just to build the rapport with nurses. It was like, everyone’s all huddled away in separate offices.

Participant 1, United Kingdom

#### Clinician Expectations

##### Mental Health Staff May Act as a Filter

As seen in the data from both the team meeting notes and focus groups, the research team was concerned that mental health staff sometimes acted as gatekeepers for some service users. This *gate keeping* behavior appeared to be expressed when mental health staff assumed a potential participant would be unable to take part in the study, resulting in a filtering effect that biases which participants are invited to take part. Trial staff constructed that the concept of gatekeeping extended beyond participating in clinical research and was perhaps linked to mental health staff feeling protective over patients in their caseload. In the following example, a researcher reflects on how mental health staff appeared to very quickly decide whether a service user could cope with the intervention:

Even when you approached them with eligible participants, they [staff] were maybe more likely to discount them straight away. Just say “no, they’re not suitable,” or “I don’t think they want to take part.”

Participant, United Kingdom

##### Mental Health Staffs’ Resistance to Research Participation

Research staff working on EMPOWER theorized that mental health staffs’ resistance to research participation emerged because mental health staff believed that they were expected to participate in clinical research as part of their role as mental health clinicians. There were some concerns that if mental health staff felt that their participation in the project was mandatory, this may have limited their motivation and commitment, resulting in resistance to participation. In the following example, a member of the EMPOWER trial reflects on an encounter with a clinician who stated that they had to become involved because of expectations from management. This appeared to be linked with hierarchical relationships within mental health services. Therefore, clinical staff participating within research appeared to be a role expectation for clinical staff:

I remember one staff member talking about whether he agreed to be involved and he said “oh, do I really have a choice?” kind of saying “well, we've heard about it from, you know, management” and I got the sense he was communicating there was an expectation to get involved but that was just one thing I picked up about that kind of involvement. Yeah.

Participant, Australia

### Trial Work Used to Facilitate Recruitment

Trial staff used several trial work strategies to facilitate recruitment in face of barriers, including flexibility in approach to barriers, persistence, and emotional labor (trial staff managing feelings and expressions to successfully recruit participants), in addition to building relationships (using preexisting relationships with clinicians and using supportive research team relationships).

#### Flexibility in Approach to Barriers

Regardless of how barriers to recruitment were negotiated, something that stood out in both the minutes and the focus groups was the need for trial staff to be flexible in their approaches. Discussions around the benefits of the flexible approach were common throughout both the Australian and UK focus groups. In the following example, a team member from Australia highlights that being flexible (and not rigid) in their approach to recruitment enabled staff to work through problems as they occurred:

I think that one of the real strengths in our research team has been how flexible and adaptive we’ve been when these challenges have come up, everyone involved in the process has been really thinking about ways to problem solve these things and coming up with suggestions.

Participant, Australia

One example trial staff provided, which illustrates taking a flexible approach, was in their discussions with clinical staff surrounding the trial protocol. Within a feasibility study, information about the recruitment process is a key outcome. Therefore, when encountering potential staff *paternalism* toward patients on their caseload, trial staff could emphasize that knowing how many people would refuse to take part was an important trial outcome. Explaining to trial staff that the protocol required that all relevant participants should have the opportunity to be approached, to discover the number of patients who did not want to take part, was described as it could circumnavigate the perceived filtering behaviors by clinical staff. In the following example, a principal investigator also describes how being flexible could enable trial staff to resist or negotiate staff paternalism, without it seeming like a direct challenge to clinical judgment:

...and our primary method of trying to get around that was to blame a third party to blame the protocol which says we needed to screen everyone and invite everyone rather than, you know directly, it feeling [sic] more like a direct challenge to the judgement of the key clinicians.

Participant, Australia

The researcher noted in their reflective memo that flexibility appeared a key process that emerged from the very beginning of recruitment when trial staff were working to build relationships and engage with the staff. Trial staff did not appear to rigidly stick to one recruitment approach:

When looking through minutes from the start of the trial. I am struck by how apparent flexibility was from the early stages of recruitment. For example, working around the availability of clinical staff as much as was possible. Furthermore, it feels important to note that because clinical staff are so busy that being flexible appeared essential in moving recruitment forward. However, in later stages flexibility involved clinical trial staff.

Researcher’s reflective memo

#### Persistence

Within EMPOWER, *trial work* was characterized not only by flexibility but also by persistence. This could be seen in accounts of trial staff constantly trying to contact mental health staff. The practical work of chasing up mental health staff was readily apparent from the analysis of the minutes of meetings and reflective accounts of the recruitment process recorded in both focus groups. Chasing up could involve telephone calls, email, or visits in person to community mental health teams. This was often because of systematic issues such as a lack of staff time to support the intervention but could also be because of local factors such as mental health staff feeling pressurized into taking part by management and resisting participation. However, linked to staff describing their need to be persistent, there was acknowledgment that chasing up mental health staff could be a time-consuming part of trial work:

It depended quite a lot on the key workers that were involved within teams. How open they were to the study, and how much they followed through on things they said they were going to do. So, a lot of the time was spent chasing up key workers who said they would do something, and then didn’t.

Participant, United Kingdom

#### Emotional Labor

Although the need to be persistent in chasing up mental health staff and trying out different recruitment strategies was apparent from both the minutes of meetings and the focus groups, the focus groups foregrounded an important role for the emotional aspects of recruitment within a clinical trial. In the following example, it is clear that simply being persistent is not enough and that it is important for it not to be obvious that the research team experienced frustration. Indeed, the need to portray constant positivity to get the work done appeared to be considered key in successfully recruiting participants. Therefore, there appeared to be an important role for *emotional labor* within trial work:

Persistence. Always smiling. Always the utmost professionalism.

Participant 1

Sometimes it’s fake. [shared laughter].

Participant 6, United Kingdom

To the best of my knowledge, no trial staff used the term emotional labour to describe the maintaining professionalism during interactions with mental health staff, carers and patients. However, when reflecting on my observations of the research process, emotional labour appeared a highly relevant interactional framework for understanding the actual work underpinning trial staff describing the competency of staying polite and professional even when faced with potentially stressful challenges. Emotional labour seemed especially pertinent because trial staff are trying to invoke positive feelings within clinical research staff to build trust in both the project and the research team themselves.

Researcher’s reflective memo

#### Relationship Building With Mental Health Staff

Trial work appeared to be sustained and facilitated by relationship building. When trial staff described the work that they performed throughout the recruitment process, at all stages, the work appeared to be underpinned by trial staffs’ ability to successfully build and use relationships. In the absence of the ability to tap into existing relationships, trial staff had to be able to quickly build working relationships with clinical staff to facilitate the recruitment process. Reflecting on the overall emergent process, trial staff centered on the importance of building relationships with clinical staff in both the United Kingdom and Australia. One key change that came from this was trial staff becoming trusted to make direct approaches to patients instead of always having to go through mental health staff:

I think the reason that it became more possible was um that the services got used to the research team and got confident in the research team, or at least management did, so I think there’s something about us building the relationship that enabled us to move into a different way of doing it.

Participant, Australia

By appraising the minutes of team meetings, it is clear that trial staff initially had to go almost entirely through mental health staff. However, if a good relationship was built, this was perceived as helpful for recruitment because the staff were generally more engaged with the team:

Within two months, trial work moved on to the establishment of relationships between mental health staff and the research team. In this stage, the EMPOWER staff became trusted to make direct approaches. Linked to the process of building relationships over time with mental health staff, in both Glasgow and Melbourne, a clinical team member [Research Nurse and Peer Support Worker, respectively] became involved in trial recruitment. Both teams reflected upon this positively because both of these clinical team members brought their pre-existing relationships with clinical staff. While the earlier stages of recruitment may have seemed slow, it appears productive in terms of carrying out trial work that built relationships and trust with clinical staff, ultimately moving trial recruitment forward.

Researcher’s reflective memo

#### Using Preexisting Relationships

Although building relationships underpinned all aspects of trial work, preexisting relationships were described as helpful in establishing clinician trust. The *trial work* here is the insight and ability of the trial staff to use those preexisting relationships in the service of recruitment. In the example given below, a research assistant stated that clinical staff felt more comfortable communicating negative feelings about the recruitment process to the peer support worker (part of the EMPOWER trial team) because of preexisting ease and trust that come with already knowing someone. The research team was then able to use this information and adapt the approach taken to recruitment to be less aversive for clinical staff:

I think the real turning point where [peer support worker who participated in recruitment process] was speaking to somebody perhaps because she has that more casual kind of pre-existing relationship with some of these people where they were explicitly saying “I’m a bit sick of this EMPOWER stuff” and that’s when you know, that sent out the message we need to pump the brakes hard in terms of how much we are asking clinicians to do here.

Participant, Australia

#### Relationship Building—Internal Within the Research Team

Relationships appeared to serve important internal functions within the EMPOWER team. Across both the United Kingdom and Australia, trial staff made reference to the importance of having team members who understood the challenges associated with clinical trial recruitment. Furthermore, the importance of having space to be open about difficulties encountered, so that discussions were focused on how best to move forward, was described:

Because I think at times it is quite demotivating. And particularly if you’ve got that third [unanswered] phone call and think “please just answer the phone.” I think we [trial recruitment staff] do try and support each other through those times.

Participant, United Kingdom

From the meeting minutes, being part of the UK meetings while recruitment was on-going and appraising themes constructed during the focus groups, it seemed as though having a space within the trial team to discuss and share frustrations that were inevitable from negotiating the various recruitment barriers. From my observations of actual meetings and continued within the focus groups, there appeared to be lots of in-jokes within the teams about the recruitment process including challenging aspects. For trial staff, this appeared to provide camaraderie and support.

Researcher’s reflective memo

To summarize, relationship building internally within the team appeared to be just as important in facilitating the recruitment process as building external relationships with mental health staff. Trial staff were there for each other throughout recruitment challenges and provided a supportive space for each other to discuss problems.

## Discussion

### Principal Findings

This study explored recruitment from the point of view of trial staff working on a digital intervention for psychosis. Detailed descriptions of the recruitment process are rarely reported within RCTs of digital interventions for psychosis, which minimizes the opportunity for sharing learning on how best to overcome recruitment barriers. By examining the recruitment process in EMPOWER using ethnography supplemented with focus groups, we now present such a detailed description. In doing so, we demonstrate not only the kind of recruitment barriers encountered by trial staff but also what strategies trial staff use to overcome them. Recruitment barriers appeared to span macro (structure and systems, eg, lack of staff time), meso (roles, eg, staff leadership), and micro (idiosyncratic, eg, the physical layout of community mental health premises) levels. The findings from this qualitative study suggest that simply reporting the number of participants recruited (n=86) clouds a highly complex social process underpinning trial recruitment. Taken together, the findings from this study can start to theorize the recruitment barriers and facilitators within the recruitment process for the EMPOWER trial.

Although it has been recommended that research exploring recruitment barriers should go beyond reporting a lack of staff time [31], it appeared a systemic problem within this trial that trial staff found difficult to negotiate. Lack of staff time has been reported as a recruitment challenge in many mental health studies [56]. Therefore, our results support those of Skea et al [54], who suggested that researchers should consider how essential trial recruitment processes fit in with the reality of clinical practice. The nonadoption, abandonment, scale-up, spread, and sustainability (NASSS) framework [57] provides a framework for understanding challenges encountered in the implementation of digital technologies. NASSS frames challenges as being simple (straightforward and predictable), complicated (multiple interacting components), or complex (unpredictable and hard to reduce down into linear components). NASSS addresses challenges and complexities that occur in different domains when implementing health care technologies, including the health condition being intervened on, value proposition, technology, adopter system, organization, wider social context, and changes over time. When framing the recruitment process via health care organizations in the United Kingdom and Australia, it appears that macrolevel recruitment barriers pose particularly complex challenges because of severe resource pressures, with staff struggling to find time to support research, as noted by other clinical trial researchers [58]. However, even more idiosyncratic challenges such as differences in leadership between cluster sites were noted by trial staff to have complex, unpredictable, and sometimes large impacts on recruitment, supporting the need to understand contextual differences across clusters in cRCTs [39].

To negotiate complex recruitment barriers, trial staff put significant amounts of work in to engaging mental health staff during the recruitment process. Trial work is multifactorial and comprises emotional labor and social and professional competencies. Initially, in performing trial work, staff in EMPOWER reported the importance of persistence, being flexible in trying different approaches, and always being professional in their interactions with staff. Previous research on clinical trial staff has suggested that emotional labor is a key part of trial work when staff are working to meet recruitment targets [59]. In the face of stresses and strains created by recruitment barriers, trial staff have a duty to maintain an ethos of professionalism. Coming from the field of sociology, emotional labor is described as the silent work of evoking feelings in others and managing one’s own emotional expressions to do so [60]. Emotional labor appeared a key strategy when dealing with barriers such as having to pursue contact with very busy staff while maintaining good working relationships by not letting frustrations show. Relationships between trial staff and clinicians (and the ability to quickly build and rapport) appeared essential to successful recruitment. However, barriers existed in the recruitment process, which could make relationship building difficult. Although a lack of clinical staff time is well reported in the literature, factors such as the layout of buildings, making it impossible to have a private conversation, also acted as a relationship building block.

Clinicians’ exclusion of people independent of trial protocol criteria is noted to be a key challenge in mental health intervention recruitment [56,61]. In the case of EMPOWER, it appeared that clinicians regularly sought to exclude participants for reasons not stated in the protocol. Trial staff were given the impression that this was because of clinical staff having concerns about a service user’s ability to cope with study participation. However, trial staff sometimes seemed able to negotiate this challenge by invoking the trial protocol and reminding staff that determining directly from service users their willingness (or not) to participate was an important outcome within a feasibility study. Mental health staff filtering what patients ended up being approached for recruitment was a key theme identified in previous research exploring barriers to recruitment to nondigital psychosis studies [62]. Excluding participants for reasons not contained in the protocol likely has implications for the replicability and robustness of research findings because the selection criteria are obscured [61] and samples likely become biased. Therefore, there is a need to learn more about why this apparent *filtering* happens (from the perspective of mental health staff), particularly in digital interventions for psychosis where little is currently known [13] and there may be assumptions about ability of people with psychosis to use technology [12].

Mental health staff have perceptions of what is required from them professionally, and these perceptions seemed to cause tension and role conflict during the recruitment process. For example, clinical staff may not feel that they have the autonomy to decline participation because participating in research is a role expectation for clinical staff. Previous oncology research has indicated that nurses involved in conducting research describe a role conflict, where duty of care to the patient can sit uncomfortably with *feeling like a salesperson* when encouraging patient participation within trials [63]. Enhancing collaborations with key stakeholders such as mental health staff is stated to be important in developing better digital interventions for psychosis [20]. Therefore, it seems pertinent to understand issues such as role conflict from the perspective of trial staff and co-design recruitment procedures around the needs of mental health staff.

Persistence and flexibility of approach were important in negotiating everything from macrolevel barriers, such as a lack of staff time, to more microlevel issues, such as community mental health center managers with different styles. One key element of the flexible approach to recruitment that emerged during the EMPOWER trial was a peer support worker (a person with their own experiences of psychosis employed to support people in their use of the intervention) advising how to approach recruitment challenges. A review concluded that patient involvement in clinical research may be associated with increased recruitment (but not retention) in clinical trials [64]. However, the mechanisms underlying this effect are still unclear. Within EMPOWER, actively transforming the peer support role to encompass involvement in recruitment was reported by trial staff to have been very useful for recruitment because the peer support role brought preexisting relationships with staff and fresh insight on how best to approach recruitment challenges. Although this may be very specific to EMPOWER, it nonetheless demonstrates that experiential knowledge and enhanced capacity for relationship building with clinical staff may be important mechanisms to consider when theorizing mechanisms of patient and public involvement in trial recruitment.

### Future Research

The research team reported that conveying to staff that highlighting the importance of gathering data on rates of participant refusal was helpful in negotiating filtering behavior by clinical staff. Future research could explore this observed phenomenon further, perhaps using relevant behavioral change theories as a theoretical framework [65]. Emotional labor in the context of clinical trials has previously been theorized in recruitment research involving direct interaction with patients [59]. However, these findings suggest that emotional labor may be relevant in the everyday work of keeping clinical staff engaged in the recruitment process. The EMPOWER trial was conducted simultaneously in Australia and the United Kingdom. Therefore, it is perhaps unsurprising that a specific recruitment issue unique to one health care system that was observed (high patient turnover within Australia) was apparent. However, there were some marked similarities across countries, such as a lack of staff time. Clinical trials conducted across multiple countries may benefit from providing some context on differences between mental health care systems to contextualize recruitment results. In addition, a Delphi study [66] could expand upon the barriers identified here to see if they are more widespread in trials of similar interventions.

### Limitations

EMPOWER was a feasibility study, which means there were a limited number of trial staff to observe and speak to. Beyond the small sample, the findings from this study should be considered in light of several key limitations. Ethnography is an opportunistic methodology [67]; therefore, researchers are limited by what they can or are allowed to observe. With regard to research methods, we did not believe that the focus group conducted remotely was any less rich than the focus group that was conducted in person in terms of the transcripts produced. However, it is important to highlight that conducting one focus group in person and another remotely may have impacted both the conduct of the research and the analysis. Moreover, although Australian recruitment was discussed at UK-based meetings and was recorded in the minutes there, SA did not attend any Australian recruitment meetings because of being based in the United Kingdom and did not directly observe Australian staff during the recruitment process. Although this study identified barriers and suggested potential ways to optimize recruitment, the potential positive impact of qualitative research in trial recruitment research needs to be further explored [35] before any comment can be made about potential utility. Furthermore, we have not focused on retention, which is also an important issue in its own right [68,69]. In addition, this study focused on barriers and facilitators experienced by trial staff during the recruitment phase of the trial and are likely biased toward their own perspectives.

Facilitators addressing ongoing *service characteristics* such as staff turnover and physical environment may have emerged if the study had been widened to include service managers or other informants. Furthermore, there was not much focus on the experiences of service user participants throughout the focus groups. Future research understanding barriers and facilitators to recruitment from the point of view of service users within clinical trials of digital interventions for psychosis, building upon previous work exploring what service users think about digital interventions for psychosis in general [70-72] and their feelings about recruitment into a clinical trial for distressing voices that involved interacting with a digital avatar [72]. Another key limitation is that recruitment within EMPOWER occurred in public mental health care systems in both Australia and the United Kingdom; however, recruitment in private health care systems or recruitment processes conducted remotely through the internet may have unique challenges. Finally, the focus of this study was to empirically explore recruitment from the point of view of trial staff; however, it is important to highlight that future research would benefit from exploring recruitment from the perspectives of clinical staff and service users, which will develop a more ecologically valid overview of the recruitment process.

### Conclusions

Exploring recruitment from the perspective of trial staff provides rich insights into barriers and facilitators to recruitment within clinical trials of digital intervention. For example, rather than people diagnosed with schizophrenia being a *hard-to-reach group*, it seems that difficulties in recruiting people diagnosed with schizophrenia to clinical trials emerge from complex dynamic interactions within health care systems. This study suggests that recruitment in a clinical trial of a digital intervention for psychosis is complex. Barriers to recruitment exist at micro, meso, and macro levels, and trial staff must negotiate these barriers within their role to meet recruitment targets to the best of their abilities. Key competencies observed during the recruitment process included flexibility, persistence, and emotional labor. As discussed in focus groups and aligned with ethnographic observations, it was important for trial staff to work within a team that understood that recruitment to clinical trials could be challenging and appreciated having access to peer support from other trial staff. People responsible for managing staff who recruit into clinical trials may wish to consider these relationship-focused factors when deciding how best to supervise staff and design effective and resilient teams. One key conclusion from this study is that learning about what works along the way is important, as it provides a space for trial staff to discuss the recruitment process and both learn from and support each other during recruitment. Relationship building with clinical staff appeared to help facilitate the recruitment process, which may have important implications for credentialing, training, and supervising staff who work within clinical trials.

## Abbreviations

CMHS: community mental health services
CONSORT: Consolidated Standards of Reporting Trials
cRCT: cluster randomized control trial
EMPOWER: Early Signs Monitoring to Prevent Relapse in Psychosis and Promote Well-being, Engagement, and Recovery
NASSS: nonadoption, abandonment, scale-up, spread, and sustainability
RCT: randomized control trial
SWAT: study within a trial
